# Excision of a Giant Bilobed Second Branchial Cleft Cyst: A Case Report

**DOI:** 10.7759/cureus.110419

**Published:** 2026-06-07

**Authors:** John W Seibert

**Affiliations:** 1 Otolaryngology - Head and Neck Surgery, Vanderbilt University Medical Center, Nashville, USA

**Keywords:** bailey classification, branchial cleft cyst, cervicotomy, congenital neck mass, giant neck mass, second branchial apparatus, spinal accessory nerve

## Abstract

Second branchial cleft cysts (BCCs) are congenital cystic anomalies of the lateral neck that rarely attain giant proportions exceeding 10 cm. When they do, their size and proximity to critical neurovascular structures present distinctive diagnostic and surgical challenges. We present the case of a 24-year-old woman with a one-year history of a painless, enlarging left neck mass. Imaging revealed a large, bilobed cystic lesion deep to the sternocleidomastoid muscle extending from the skull base to the subclavicular region. Fine-needle aspiration cytology excluded malignancy, and the cyst recurred after ultrasound-guided aspiration. The patient underwent complete surgical excision with preservation of the spinal accessory nerve and all adjacent neurovascular structures. The postoperative course was uncomplicated, and pathology confirmed the diagnosis. This case illustrates that second BCCs can attain extraordinary dimensions and exhibit complex morphology while remaining benign. A multimodal preoperative workup is essential to exclude malignancy and guide surgical planning. Controlled intraoperative decompression can facilitate safer dissection in unusually large lesions. Complete surgical excision remains the definitive treatment and is critical for preventing recurrence and complications.

## Introduction

Second branchial cleft cysts (BCCs) are the most common congenital cystic lesions of the lateral neck, accounting for approximately 67-93% of all branchial apparatus anomalies [1,2]. They arise from incomplete obliteration of the cervical sinus of His during embryogenesis, when the second pharyngeal arch normally overgrows the second through fourth branchial clefts between the fifth and seventh weeks of fetal life [3]. Clinically, these cysts typically present as painless, smooth, fluctuant masses at the anterior border of the SCM in the second through fourth decades of life [4].

Bailey’s classification describes four anatomical subtypes based on depth and relation to the carotid vessels, with Type II, located beneath the middle cervical fascia in the prevascular region, being the most frequently encountered [5]. Most second BCCs range from 1 to 10 cm in maximum diameter, whereas lesions exceeding 10 cm are classified as “giant” variants and are uncommon, often posing unique operative challenges [6,7].

Complete surgical excision is the universally recommended treatment, as incomplete removal risks recurrence, and untreated lesions carry risks of infection, compression of adjacent structures, and rare malignant transformation [8]. Preoperative evaluation with cross-sectional imaging (CT or MRI) and fine-needle aspiration cytology (FNAC) is essential to characterize lesion extent, exclude malignancy, and guide operative planning. When these lesions attain a large or bilobed configuration, they may displace or envelop critical neurovascular structures, including cranial nerves and the carotid sheath, substantially increasing operative complexity.

We present a case of a giant bilobed second BCC in a young adult female, with a cyst extending from the skull base to the subclavicular region, managed by complete surgical excision with a nerve-sparing technique. This report was prepared in accordance with the Surgical CAse Report (SCARE) 2023 reporting guidelines [9].

## Case presentation

A 24-year-old woman with a past medical history of asthma presented to her primary care physician with a two-month history of a painless mass on the left side of her neck. The patient reported that the mass had been noted by others rather than discovered by herself. She denied dysphagia, odynophagia, voice changes, shortness of breath, fever, night sweats, or unintentional weight loss. She was a lifetime nonsmoker with no relevant family history of head and neck disease. Her prior surgical history was limited to a previous dental procedure.

Physical examination revealed a well-appearing female in no acute distress. An approximately 11 cm, non-tender, soft, cystic mass was palpated in the left lateral neck. The overlying skin was unremarkable. No cervical lymphadenopathy was identified. Cranial nerves II-XII were grossly intact.

Diagnostic workup

**Figure 1:**
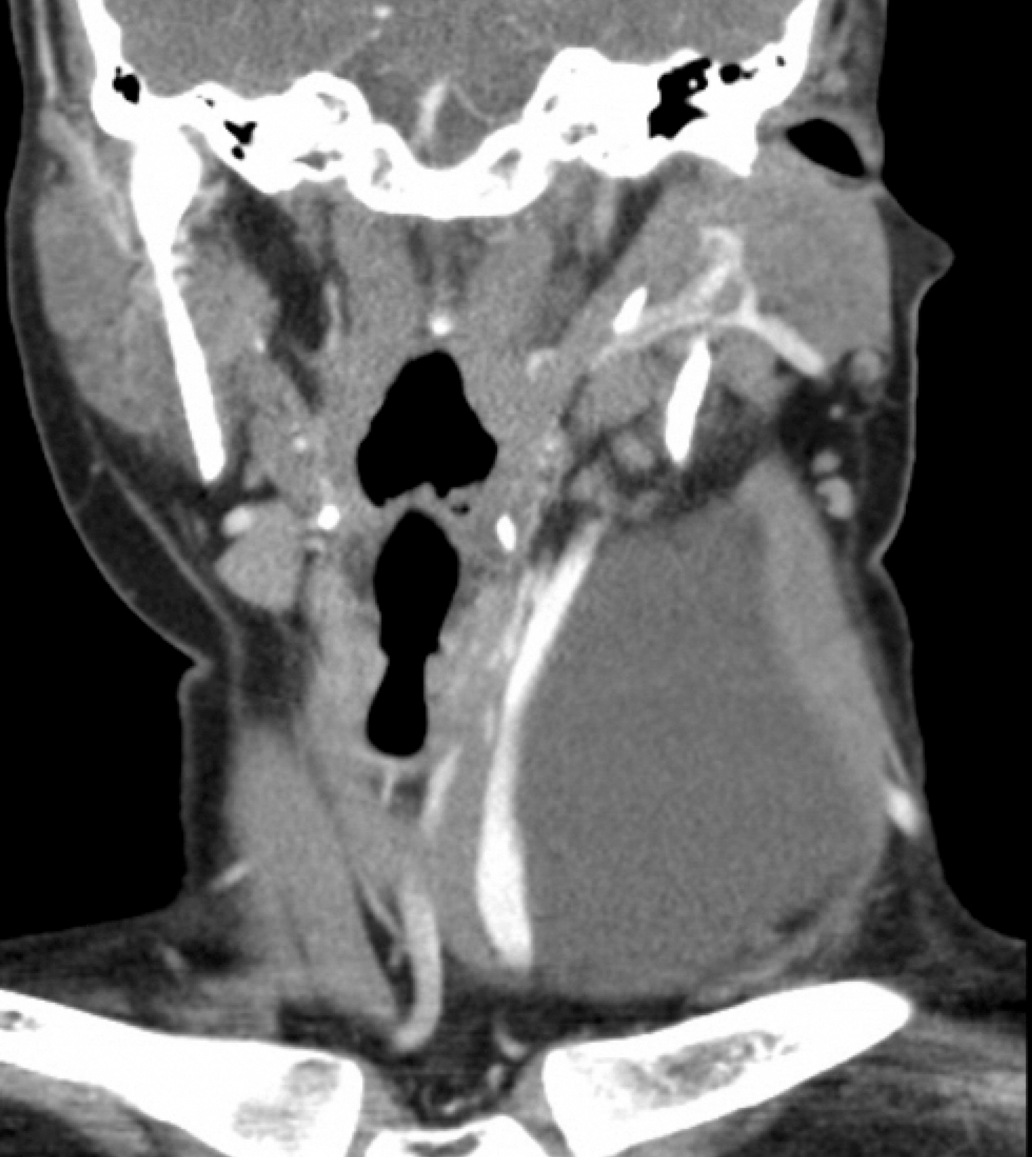
Coronal CT with contrast of BCC splaying the internal jugular vein anteriorly and medially BCC, branchial cleft cyst

Contrast-enhanced CT of the neck demonstrated a large cystic mass on the left side measuring 8.3 × 12.2 cm, located deep to the SCM (Figure 1). The oropharynx, hypopharynx, larynx, trachea, retropharyngeal space, salivary glands, and thyroid gland were unremarkable. No lymphadenopathy was identified. The CT differential diagnosis included BCC, cystic hygroma, lymphangioma, laryngocele, cervical bronchogenic cyst, and ranula.

The patient was referred sequentially to general surgery and then to otolaryngology-head and neck surgery. Ultrasound-guided aspiration of the cyst was performed, resulting in complete radiographic flattening of the lesion. A cytospin specimen from the aspirate was sent to surgical pathology; the cytopathology report (verified by the attending pathologist) showed a scant number of benign epithelial cells with no malignant cells identified, consistent with a benign cystic process. The cyst recurred within approximately two weeks of aspiration, prompting referral for definitive surgical management.

**Figure 2:**
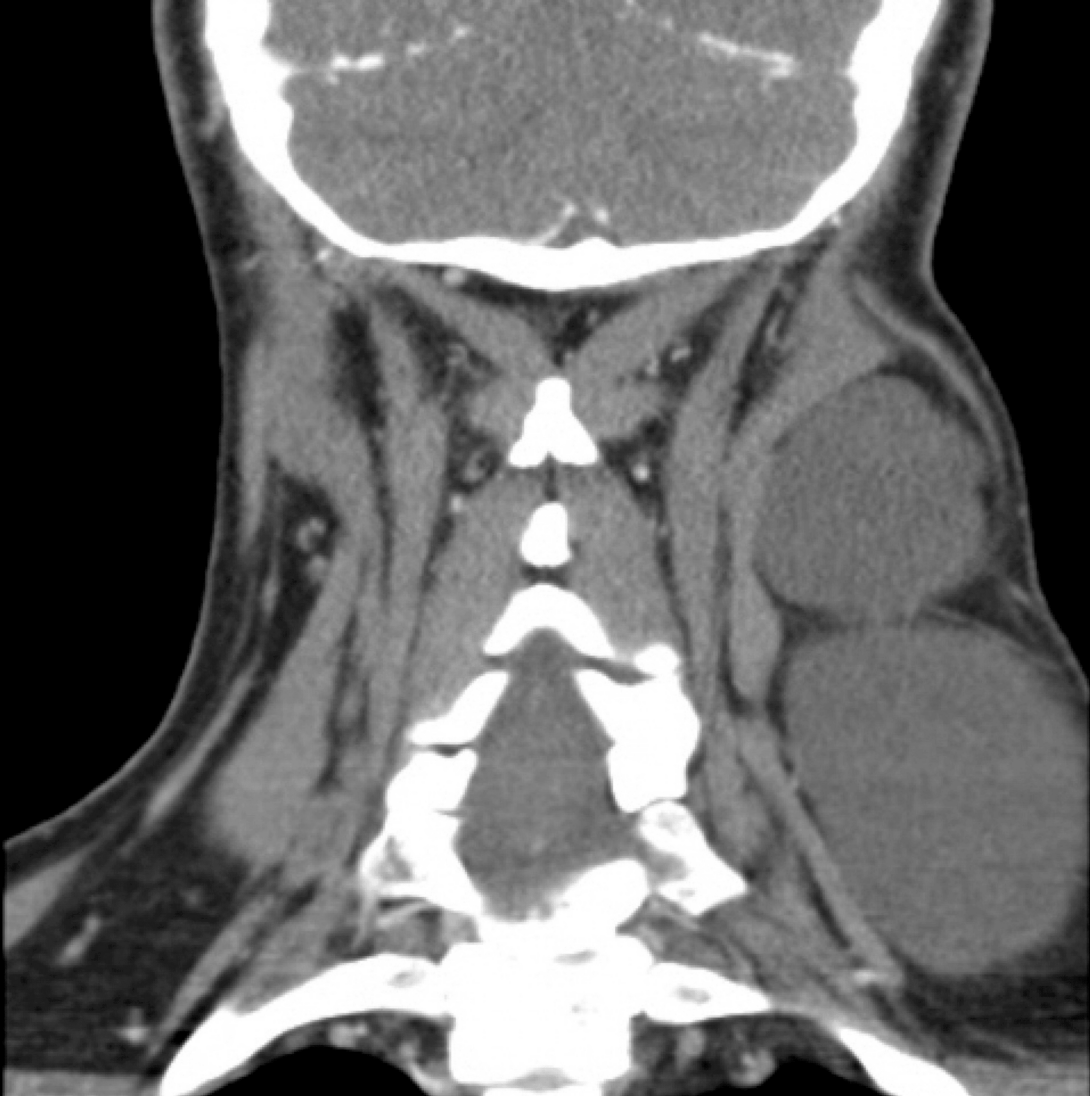
Bilobed BCC split by CN XI BCC, branchial cleft cyst

On examination by the ENT surgeon, a large, non-tender, soft neck lesion was again confirmed on the left. Review of CT (Figure 2) and cytology results confirmed the working diagnosis of a left second BCC in a bilobed configuration that was anatomically separated by CN XI.

The surgeon discussed the CT findings and recommended surgical excision, reviewing all operative risks, including injury to adjacent neurovascular structures. The patient consented to operative intervention, which was scheduled to allow time for financial assistance arrangements.

Operative management

Preoperative Diagnosis

**Figure 3:**
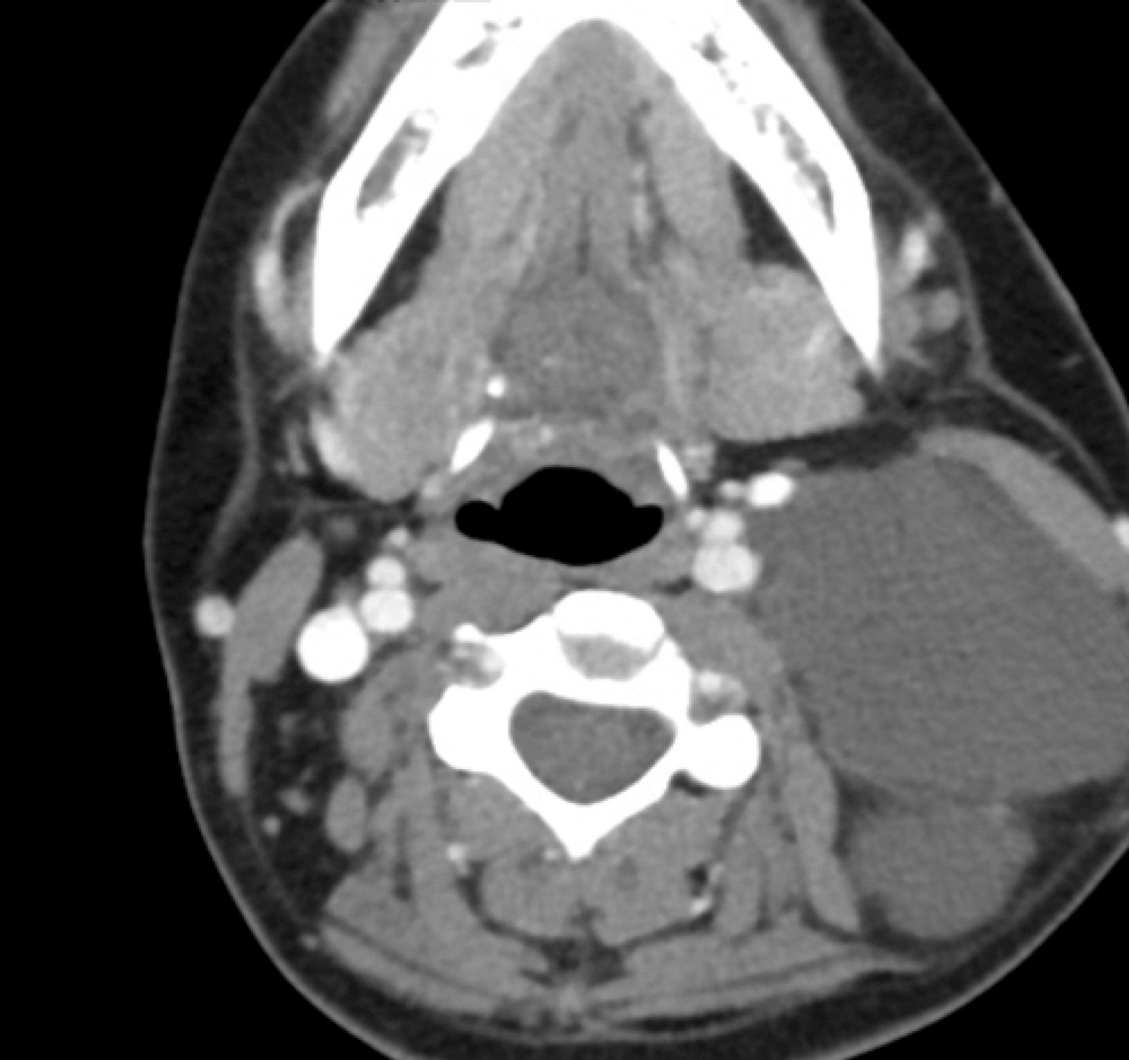
Axial CT scan of the neck

A complex bilobed BCC of the left neck was identified, extending from the skull base to the subclavicular region and measuring approximately 12 × 8 cm (Figure 3).

Surgical Procedure

The patient was placed supine under general endotracheal anesthesia. The head was turned to the right, and a shoulder roll was placed beneath the left shoulder to extend the neck and optimize cervical exposure. The left neck, face, and upper chest were prepped with chlorhexidine and draped in standard sterile fashion. Subcutaneous lidocaine 1% (10 mL) was infiltrated along the planned incision for local analgesia and vasoconstriction.

**Figure 4:**
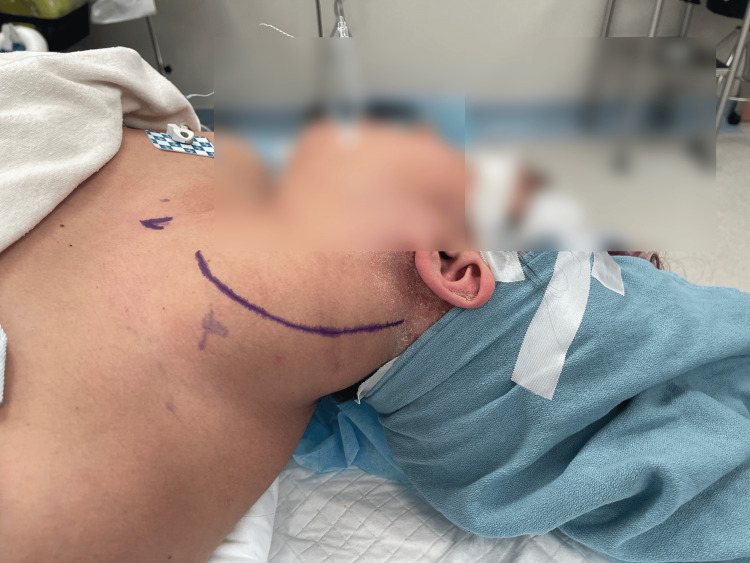
Preoperative incision

A curvilinear skin incision (Figure 4) was made along a natural skin crease of the left neck, oriented to allow adequate superior and inferior exposure of the entire bilobed mass from the skull base to the subclavicular region.

Subplatysmal flaps were elevated superiorly and inferiorly using electrocautery and blunt dissection. The SCM was identified and mobilized laterally. The investing layer of the deep cervical fascia was incised, and the dissection plane was developed around the cystic mass.

**Figure 5:**
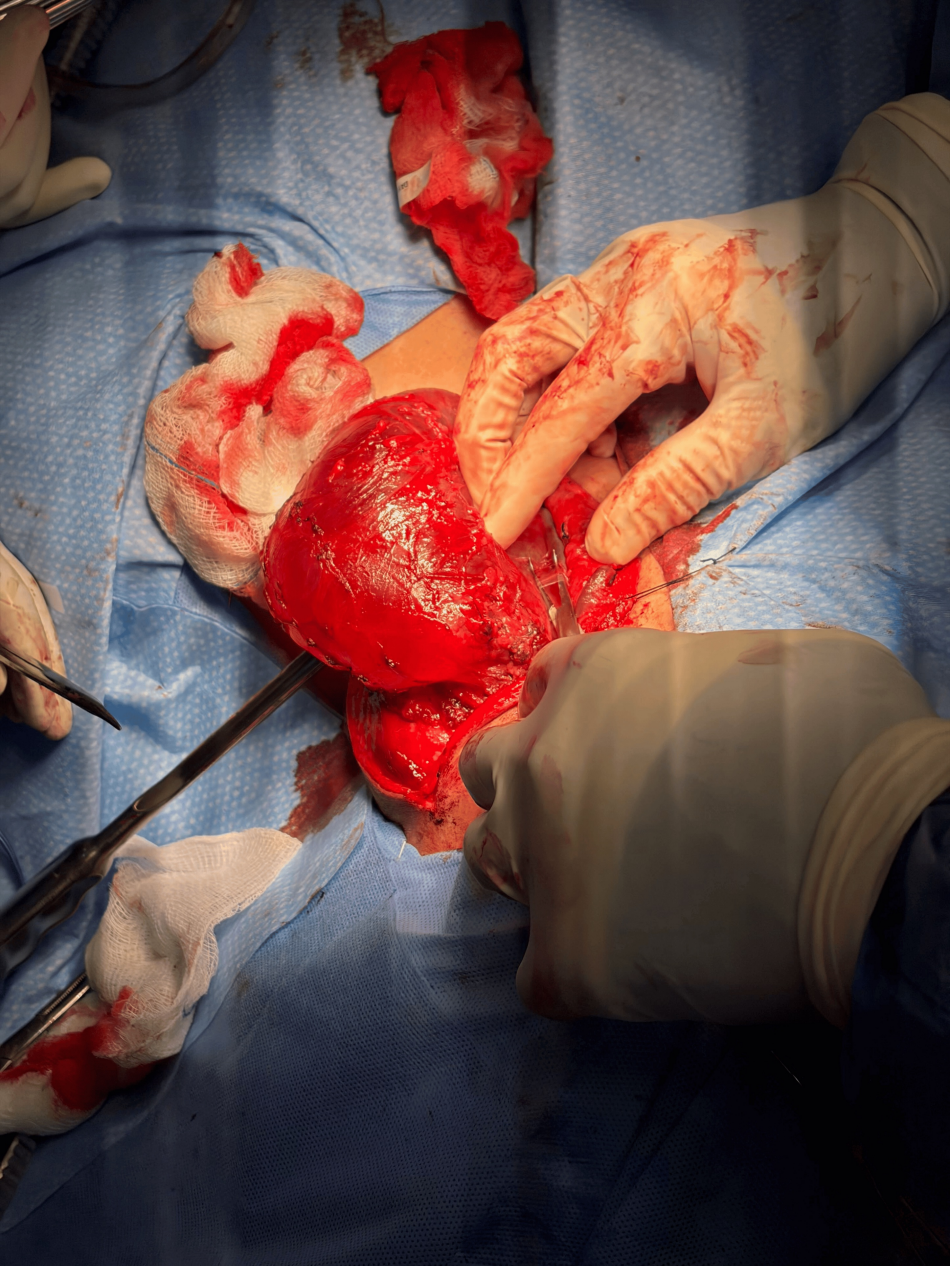
Gross surgical view of BCC BCC, branchial cleft cyst

The mass was encountered immediately upon entry into the deep cervical space as a large, tense, bilobed cystic structure with a smooth outer wall (Figure 5). The inferior lobe occupied the mid and lower neck; the superior lobe extended deep to and above the level of CN XI toward the skull base.

Given the size of the mass (≈12 × 8 cm), controlled decompression was performed under direct visualization using a large-bore needle, yielding a significant volume of straw-colored cystic fluid. Decompression caused collapse of the cyst wall, substantially improving access to the deep dissection planes.

**Figure 6:**
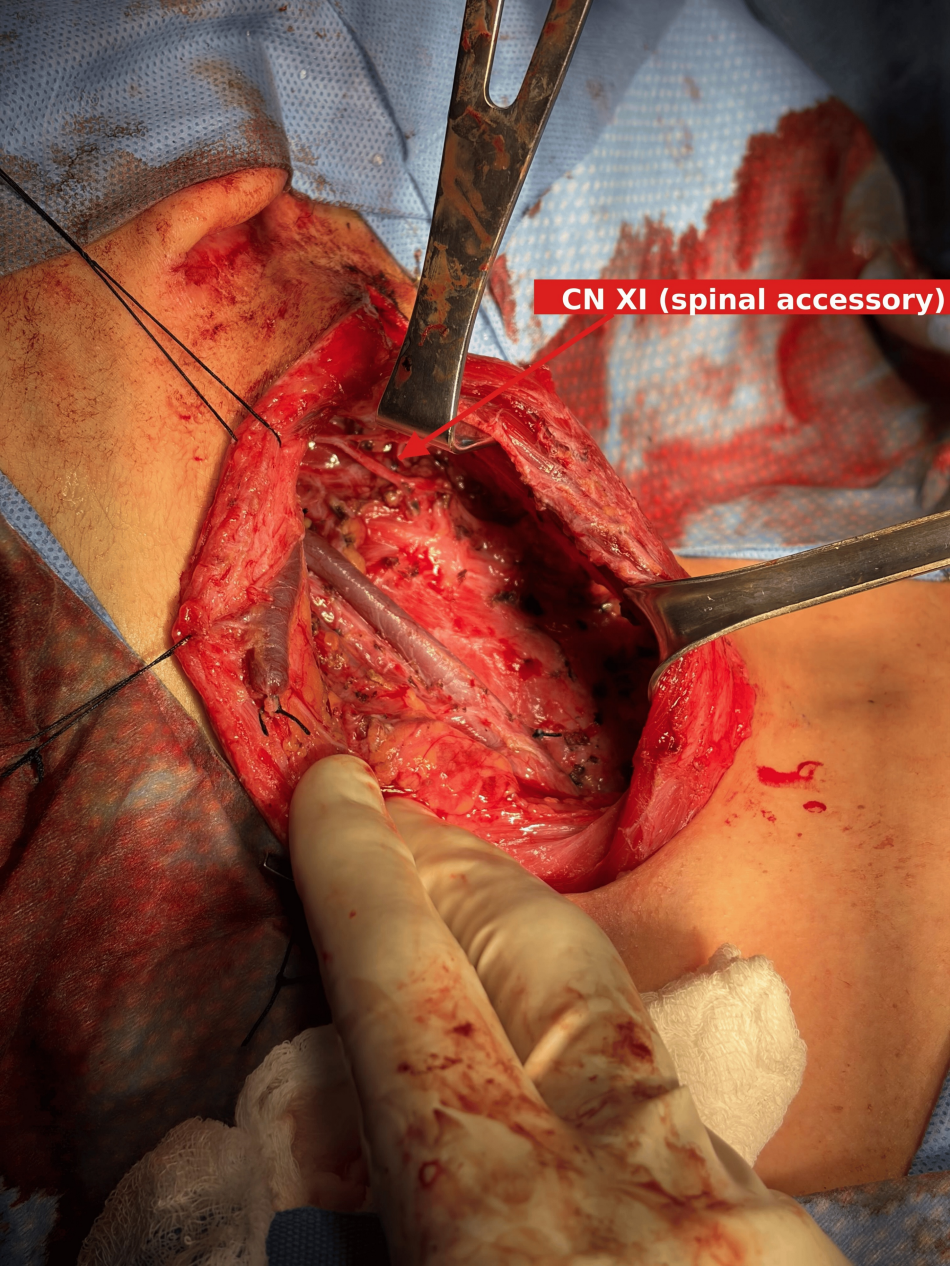
CN XI preservation after branchial cleft excision

With the cyst decompressed, CN XI (Figure 6) was carefully identified within the posterior triangle, traced from its emergence at the posterior border of the SCM to its entry into the trapezius, and protected with a vessel loop throughout.

The superior lobe was then dissected toward the skull base, taking care to avoid injury to the jugular foramen structures, the posterior belly of the digastric muscle, the styloid process, and the adjacent neurovascular bundle, including the internal carotid artery, internal jugular vein, and cranial nerves IX, X, and XII. The superior pedicle was ligated and divided.

The inferior lobe was then dissected inferiorly along the carotid sheath, with particular attention to avoiding injury to the thoracic duct on the left and the roots of the brachial plexus. The inferior pedicle was ligated and divided. The two communicating lobes were excised together as a single specimen measuring approximately 12 × 8 cm.

The wound was copiously irrigated and inspected. CN XI was confirmed intact. No active bleeding, chyle leak, or air leak was identified. A flat 10 French closed-suction drain was placed through a separate stab incision and secured. The wound was closed in anatomic layers (platysma with 3-0 Vicryl, subcutaneous with 3-0 Vicryl, and skin with a running 4-0 Monocryl subcuticular suture). Bupivacaine 0.5% (10 mL) was infiltrated peri-incisionally. The patient was awakened without difficulty and transferred to the post-anesthesia care unit in stable condition.

Specimen and Pathological Findings

The excised bilobed specimen measured approximately 12 × 8 cm in aggregate. On gross examination, the cyst demonstrated two communicating lobes with a smooth, glistening external surface and thin, pliable walls. The specimen was opened on the back table and contained a large volume of straw-colored to slightly turbid cystic fluid, consistent with the intraoperative aspirate obtained during controlled decompression. No solid nodules, calcifications, internal septations, intraluminal vegetations, or areas of wall thickening were identified grossly. The external cyst surface was smooth without evidence of capsular invasion or adherence to non-cystic tissue. The specimen was submitted in its entirety to the surgical pathology department for permanent section analysis.

On microscopic examination, the cyst wall demonstrated the characteristic histopathological features of a second BCC. The luminal surface was lined by stratified squamous epithelium without cytological atypia, dysplasia, or malignant change. No keratinization or keratin pearl formation was identified within the lumen. The subepithelial connective tissue stroma contained abundant lymphoid tissue organized into well-formed follicles with active germinal centers, a hallmark feature of BCC pathology. The fibrous cyst wall was of uniform thickness, with no areas of necrosis or hemorrhage. No evidence of malignant transformation, carcinoma in situ, or invasive carcinoma was identified. No salivary gland tissue, thymic tissue, or parathyroid tissue was seen within the cyst wall. The final pathological diagnosis confirmed a benign second BCC without evidence of malignancy.

These histopathological findings are consistent with the well-described microscopic appearance of second BCCs in the published literature. The characteristic squamous epithelial lining with subepithelial lymphoid follicles represents the most common histological pattern, reflecting the cyst’s origin from the cervical sinus of His and its intimate developmental relationship with the second pharyngeal pouch, which gives rise to the palatine tonsil [2]. Some authors consider the simultaneous presence of keratin and organized lymphoid tissue as obligatory diagnostic criteria for the BCC diagnosis, distinguishing it from other cystic neck lesions such as dermoid cysts, thyroglossal duct cysts, and mucous retention cysts [1]. Respiratory-type (ciliated pseudostratified columnar) epithelium may also line portions of the cyst wall, particularly in cases arising more proximally along the branchial embryological tract, and mixed squamous-respiratory epithelial patterns have been described [2]. The absence of intraluminal vegetations, solid mural nodules, and cytological atypia in the present specimen provided pathological confirmation that the lesion was entirely benign, consistent with the preoperative FNA findings of benign epithelial cells without malignant features.

Postoperative course

The patient recovered uneventfully. The closed-suction drain was maintained until output fell below 30 mL per 24-hour period, then removed in the clinic. Follow-up was scheduled within seven to 10 days of surgery, with assessment of shoulder function and range of motion to confirm intact CN XI function. Final surgical pathology confirmed a benign second BCC without evidence of malignancy, and results were reviewed with the patient at the first postoperative visit. No intraoperative or immediate postoperative complications were documented.

## Discussion

Literature review

Embryology and Pathogenesis

The branchial apparatus forms during the fourth week of gestation, consisting of four paired mesodermal arches separated by ectodermal clefts externally and endodermal pouches internally [2]. Each arch contains a cartilaginous skeleton, musculature, a cranial nerve, and an associated artery. Between the fifth and seventh weeks, the ventral aspect of the second arch enlarges caudally to overgrow the second, third, and fourth clefts, forming an ectodermal depression known as the cervical sinus of His [2]. By the seventh week, this sinus and the second through fourth grooves normally involute completely, giving the neck a smooth contour.

Second BCCs arise from the failure of complete obliteration of the cervical sinus and its duct during this developmental process [1,2]. The resulting entrapped epithelium forms a cyst lined by squamous or respiratory epithelium, typically situated in the deep cervical compartment. The branchial apparatus gives rise to many critical head and neck structures: the first pouch forms the eustachian tube, middle ear, and mastoid antrum; the second pouch develops into the tonsillar fossa; and the third and fourth pouches contribute to the thyroid and parathyroid glands and thymus [2].

Although branchial remnants, cervical sinus remnants, and thymopharyngeal duct remnants are all proposed as possible origins of lateral cervical cysts, the prevailing view in contemporary literature regards these lesions as congenital branchial remnants [9]. Daoud’s retrospective series of 34 branchial cysts noted that the vast majority originate from the second branchial cleft and pouch remnants and are found in the classical anatomical position anterior to the SCM [9]. A minority of theories suggest that cystic metaplasia of cervical lymph node epithelium may account for some cases, as up to 96% of specimens contain well-differentiated lymphoid tissue [2].

Epidemiology and Clinical Presentation

Second BCCs represent the most common branchial apparatus anomaly, comprising 67-93% of all cases in the most frequently cited series [1-3]. They account for approximately 2% of all laterocervical tumors and 17% of pediatric cervical masses [9]. Among patients with cervical swelling, branchial anomalies have been identified in up to 17% of cases [4]. Second cleft anomalies are eight to 10 times more common than first cleft anomalies, while third and fourth cleft anomalies are quite rare [2].

The lesions most commonly present between the second and fourth decades of life, with a mean age at presentation reported as approximately 20-30 years across multiple series [1,9,10]. Bellakhdhar et al., in a 14-year retrospective study of 34 second BCCs, reported a mean age of 27 years with a range of 4 to 74 years [1]. Daoud’s Jordan series of 33 patients reported a mean age of 20 years with a range of 1 to 57 years [9]. Notably, Howlett et al. described what they believed to be the oldest reported case of an isolated parapharyngeal BCC in a 70-year-old male, underscoring that age should not exclude this diagnosis [3]. Similarly, Najib et al. reported a 70-year-old woman presenting with a right supraclavicular branchial cyst [7].

There is no firmly established sex predominance. Bellakhdhar et al. reported a sex ratio of 0.8 (16 males, 18 females) [1], while Daoud’s series included 22 females and 11 males [9]. The Houas pediatric series noted a marked female predominance of 80% [10]. Bilateral second BCCs are rare, with a reported frequency of approximately 2% in the literature, and their presence warrants screening for branchio-oto-renal (BOR) syndrome [1,10].

The classical clinical presentation is a painless, smooth, compressible, slowly enlarging mass at the anterior border of the SCM at the junction of its upper and middle thirds [2,3,9]. Bellakhdhar et al. found that 77% of their 34 cases presented with a jugulocarotid localization, while 6% were supraclavicular [1]. The cyst is typically described as renitent or elastic, round to ovoid, mobile with respect to superficial and deep planes, and covered by normal overlying skin in the absence of infection [1]. Acute enlargement may occur following an upper respiratory tract infection; three patients (9.1%) in Daoud’s series noted enlargement with respiratory infections, consistent with earlier reports [9].

In larger lesions, compressive symptoms may develop, including dysphagia, dysphonia, dyspnea, tracheal deviation, neck asymmetry, and even bradycardia from carotid bulb compression [5]. El Omri et al. reported a 31-year-old woman with an 8 cm left second BCC extending from the hyoid bone to the supraclavicular region with no compressive symptoms despite its size [5]. Berrerhdoche et al. described a 33-year-old man with a 65 × 50 mm right second BCC causing extrinsic compression of the anterior surface of the internal jugular vein, also without compressive symptoms [6].

Diagnostic Evaluation

Imaging: Cervical ultrasound is the recommended first-line imaging investigation for lateral neck cysts. It provides structural analysis of the mass, confirms cystic versus solid character, and may reveal internal features such as echogenic particles, thick walls, or internal septations that indicate complicated or infected cysts [1,10]. The typical sonographic appearance is a well-defined, unilocular, anechoic mass without internal septation; however, secondary infection and hemorrhage may produce a more heterogeneous pseudo-solid appearance that can complicate diagnosis [9]. Bellakhdhar et al. reported that cervical ultrasound performed in 30 of their 34 patients demonstrated a cystic mass in 86.7% of cases [1]. Houas et al. used ultrasound as the first-line examination in all 10 pediatric patients in their series, identifying cystic formations in eight of 10 cases [10].

CT with intravenous contrast remains the imaging modality of choice for surgical planning, providing precise delineation of lesion size, extent, and relationships to the carotid space, SCM, and deep cervical compartment [5,10]. On CT, uncomplicated second BCCs appear as well-circumscribed, homogeneous, hypodense cystic lesions with thin walls and peripheral enhancement; wall thickening may occur after infection [11]. CT also helps identify the cyst’s relationship to adjacent vascular structures, which is essential for operative planning in large or deeply situated lesions [1]. In the present case, CT clearly demonstrated the 8.3 × 12.2 cm cyst lying deep to the SCM, enabling preoperative appreciation of its extent.

MRI is particularly useful in evaluating lesions of atypical location or parapharyngeal extension, providing superior soft tissue contrast and delineation of vascular relationships without ionizing radiation [3,6]. On MRI, second BCCs characteristically show hypointensity on T1-weighted sequences and hyperintensity on T2-weighted sequences, with peripheral wall enhancement following gadolinium injection [1,6]. Berrerhdoche et al. found that MRI confirmed the cystic nature of a huge right second BCC and its proximity to the internal jugular vein, while associated lymph nodes in territories IIa and IIb were also identified [6]. Bocchialini et al. described MRI findings of a rapidly growing 7 × 4 cm second BCC showing hyperintense content on T2-weighted sequences with well-defined margins and no infiltration of surrounding structures [11]. In cases where CT findings are equivocal or when vascular malformation must be excluded, MRI with dynamic contrast-enhanced sequences offers complementary diagnostic information.

FNAC: FNAC is an important adjunct in the preoperative evaluation of second BCCs, particularly in adults, where malignancy must be actively excluded. The cytological criteria for BCC diagnosis include (a) thick, yellow to pus-like fluid; (b) anuclear keratinizing cells; (c) squamous epithelial cells of variable maturity; and (d) a background of amorphous debris [7]. Cholesterol crystals in the aspirate are also a helpful, though not invariably present, cytological finding, identified in approximately half of cases in some series [9].

Daoud performed FNAC in 22 of 33 patients and found that benign squamous epithelial cells were present in 13 of 14 cytological examinations, highlighting the diagnostic value of identifying epithelial cells in lateral neck aspirates [9]. Bellakhdhar et al. performed aspiration in 13 of their 34 patients, with purulent fluid in 46.1% and citrine-yellow fluid in 15.4% of cases, though cytological examination was not performed in all cases [1]. Bocchialini et al. confirmed the BCC diagnosis preoperatively by FNAC, which yielded pus-like fluid with keratinized anuclear cells, squamous epithelium, and amorphous debris, allowing surgery to proceed without prior exploratory biopsy [11].

FNAC is not, however, a substitute for definitive histopathological examination of the excised specimen [7]. In the adult age group, any lateral neck cyst aspirate showing atypical or malignant cells must prompt a thorough workup for primary head and neck carcinoma with cystic metastasis, as this entity can be morphologically indistinguishable from malignant transformation within a BCC on cytology alone.

Differential diagnosis: The differential diagnosis of a lateral cystic neck mass is broad. Daoud’s series documented that only 41.2% of branchial cysts were correctly diagnosed on initial presentation, with the most common misdiagnoses being cervical abscess (45%), neoplasm (20%), tuberculosis (10%), and toxoplasmosis (10%) [9]. This diagnostic inaccuracy led to inappropriate incision and drainage in five patients, all of whom experienced recurrence [9]. These data reinforce the importance of maintaining a high clinical index of suspicion for BCC in patients with lateral neck masses, regardless of whether the mass appears cystic or solid, painful or painless.

Key entities in the differential diagnosis include cystic hygroma (lymphangioma), thyroglossal duct cyst, dermoid cyst, cervical abscess, metastatic lymphadenopathy, lymphoma, and parotid lesions [3,7,9]. Cystic hygroma, as illustrated by Shrestha et al.’s report of a 13 × 12 × 11 cm giant cystic hygroma in a 25-year-old male, is typically multiloculated, transspatial, and lined by endothelium rather than squamous epithelium and is most common in the posterior triangle [12]. In contrast, second BCCs are unilocular (unless complicated), lined by squamous or respiratory epithelium, and located anterior to the SCM [5].

Thyroglossal duct cysts occupy the midline, move with deglutition and tongue protrusion, and are closely associated with the hyoid bone, distinguishing them from laterally placed BCCs [13]. Benfadil et al.’s report of a 6.3 cm giant thyroglossal duct cyst in a 37-year-old woman illustrates how large midline cysts may occasionally be confused with branchial anomalies when they displace anatomy and emphasizes CT’s role in precisely localizing lesion origin [13]. Dermoid and epidermoid cysts differ in their CT characteristics (fat attenuation values and/or diffusion restriction on MRI), and parapharyngeal salivary neoplasms are typically solid or heterogeneous.

For the present case, the strictly lateral location deep to the SCM, CT characteristics of a thin-walled unilocular cyst without solid components, and benign FNA cytology reliably supported a second BCC over the other entities in the differential. The CT report appropriately included cystic hygroma and lymphangioma in the differential, but ultrasound-guided aspiration yielded straw-colored fluid (rather than lymph) and benign epithelial cells on cytology, further supporting the BCC diagnosis.

Bailey Classification and Anatomical Considerations

Bailey’s classification, originally described in 1929 and subsequently refined by Proctor in 1955, remains the most widely used anatomical framework for second BCCs [2,4]. Type I cysts are the most superficial, lying deep to the platysma anterior to the SCM. Type II, the most common type, representing the majority of cases across published series, lies deep to the cervical fascia in the prevascular region, medial to the SCM and lateral to the carotid sheath. Type III cysts pass between the internal and external carotid arteries, extending toward the pharynx. Type IV cysts, the rarest type, are located in the pharyngeal mucosal space adjacent to the palatine tonsil, medial to the carotid axis, and may extend toward the skull base [4].

In the Alshihmani case report, a Type III cyst was excised in a 17-year-old male, with the tract located between the external and internal carotid arteries and ligated at surgery [4]. The Houas pediatric series classified seven of 10 cases as Bailey Type II, two as Type III, and one as Type I, consistent with the predominance of Type II described in the broader literature [10]. Bocchialini et al.’s case of rapid-onset second BCC was determined to be Type II based on intraoperative and imaging findings [11]. In the present case, the superior extension of the cyst toward the skull base, with the superior lobe located deep to and superior to CN XI, is consistent with a large Type II lesion with atypical superior extension, though features of Type IV cannot be entirely excluded given the cyst’s superior anatomical reach.

The embryological tract of second branchial remnants is well defined: it extends from an external opening along the anterior border of the SCM, passes between the internal and external carotid arteries, courses superficial to cranial nerves XI and XII, passes deep to the posterior belly of the digastric and stylohyoid muscles, and terminates at the posterior aspect of the tonsillar fossa [2]. A complete second branchial fistula traverses this entire tract; cysts may occur anywhere along this course. The proximity of this tract to multiple critical neurovascular structures makes anatomical knowledge of the embryological pathway essential for safe surgical dissection.

Surgical Management

Indications and timing: Complete surgical excision is the universally recommended definitive treatment for second BCCs, given the risks of recurrent infection, progressive enlargement, and rare malignant transformation [1,5,7]. Surgery should be performed electively, after any active infection has been fully resolved with antibiotic therapy [5,10]. Bellakhdhar et al. performed surgery at a distance from infectious episodes in all four cases with superinfected cysts, a principle that is strongly supported across the literature to minimize perioperative complications and reduce the risk of incomplete excision due to inflammatory scarring [1].

The operative indication is formal once the diagnosis is established [1]. While some authors have proposed sclerotherapy as a conservative first-line option after malignancy exclusion, and others have described marsupialization for parapharyngeal variants, surgical excision remains the gold standard for second BCCs due to its superior long-term cure rates [1]. Aspiration alone is not curative; as demonstrated in the present case, the cyst recurred within approximately two weeks of ultrasound-guided aspiration, which is consistent with the universal expectation of cyst recurrence following simple drainage without complete excision.

Operative technique: The standard approach for second BCC excision is a transverse cervicotomy with a curvilinear incision placed in a natural skin crease [2]. Houck’s technical review emphasizes that incisions should follow Langer’s lines, generally placed directly over the midpoint of the cyst, and that vertical incisions should be avoided due to unfavorable scarring [2]. For larger lesions or those with significant superior or inferior extension, a McFee (stair-step) incision may be used to extend exposure while maintaining cosmetic acceptability [2].

Subplatysmal flaps are elevated superiorly and inferiorly, and the SCM is mobilized laterally to expose the deep cervical compartment. The cyst is then dissected from the superficial layer of the deep cervical fascia. Houck recommends that dissection proceed medially with careful avoidance of the carotid sheath and ansa hypoglossi, with transection of the facial vein if necessary [2]. The cyst is dissected off the posterior belly of the digastric and stylohyoid muscles, and the hypoglossal nerve (CN XII) and overlying veins are carefully preserved. If present, a fistulous duct passes between the internal and external carotid arteries; its terminal end near the tonsillar fossa must be identified and ligated [2].

Methylene blue injection of a fistulous tract has been recommended by multiple authors to facilitate identification of the tract during dissection and ensure complete epithelial removal [1,2,10]. If the fistula extends superiorly beyond the level of the initial incision, a second “stepladder” or “McFee” incision is required to safely follow and ligate the superior extent [2]. Houas et al. found that high ligation of the fistula without ipsilateral tonsillectomy was sufficient to prevent recurrence in their pediatric series, with no recurrences observed over a mean follow-up of 24 months, supporting the approach of complete excision without routine tonsillectomy [10].

For giant BCCs, controlled intraoperative decompression prior to attempted en bloc excision is a key technical maneuver described in several case reports of large lesions [5,6]. El Omri et al. noted that their 8 cm cyst required careful dissection to preserve adjacent vascular and nervous structures and that strap muscles adherent to the cyst due to prolonged compression had to be partially excised [5]. Berrerhdoche et al. performed a cervicotomy with complete cyst resection in their patient with a 65 × 50 mm lesion causing jugular vein compression, with a simple postoperative course [6]. The present case extended this principle further, with controlled aspiration of a 12 × 8 cm bilobed cyst prior to dissection, substantially improving operative visualization.

Partial aspiration of large cysts to facilitate dissection is also endorsed by Houck, who notes that partial aspiration of some cysts may help dissection and that traction on the cyst wall with forceps must be gentle to avoid rupture during excision [2]. Rupture of the cyst intraoperatively, while not always avoidable, may increase the risk of incomplete excision of the cyst wall epithelium and subsequent recurrence.

Nerve preservation: Preservation of adjacent cranial nerves is a central objective of the second BCC excision. In the posterior cervical triangle, CN XI (spinal accessory nerve) is at particular risk during dissection of lesions with posterior triangle or superior extension. Injury to CN XI produces ipsilateral shoulder dysfunction due to trapezius denervation, representing one of the most significant functional complications of cervical surgery. Formal identification of CN XI, from its emergence at the posterior border of the SCM to its entry into the trapezius, is essential whenever the dissection approaches the posterior triangle.

CN XII (hypoglossal nerve) is also vulnerable during medial dissection. Houck notes that the cyst or its tract crosses CN XII posteriorly and that indiscriminate clamping due to bleeding can easily injure CN XII; veins overlying the nerve must be carefully dissected or transected [2]. The glossopharyngeal nerve (CN IX) is at risk near the pharyngeal attachment of the fistula, while the superior laryngeal nerve courses obliquely behind the external carotid and is at risk during high dissection [2].

Howlett et al.’s case of a 70-year-old man with an unusual parapharyngeal second BCC illustrates the added complexity when the cyst extends into the parapharyngeal space: careful transoral dissection was required, and the capsule was found adherent to residual tonsillar tissue and extending into the parapharyngeal fat, with the posterior capsule plane resting on the prevertebral fascia [3]. Houck emphasizes that dysfunction of CN XII is usually transient when encountered and that recurrences requiring reoperation may necessitate functional neck dissection [2].

Complications and Recurrence

The most common complication of the second BCC excision is recurrence. Houck’s review of a large series of 208 cases reported recurrence rates of 21% in patients with prior surgery, 14% in those with prior infection, and only 3% in patients with neither prior surgery nor infection [2]. Daoud’s series confirmed this pattern: all five patients who underwent incision and drainage prior to definitive excision experienced recurrence, while no patient who underwent primary complete excision had recurrence [9]. These data underscore that incision and drainage are not only non-curative but also actively worsen outcomes by inducing scarring that complicates subsequent definitive surgery.

Bellakhdhar et al. observed a 3% recurrence rate (one of 34 patients) in a patient who had undergone excision of a cyst with a fistulous tract; the recurrence was attributed to incomplete excision of the fistula due to inflammatory changes during dissection [1]. El Omri et al. reported no recurrence at 24-month follow-up in their patient with an 8 cm giant BCC [5]. Alshihmani reported no recurrence at 14-month follow-up [4].

Additional complications include hematoma or seroma formation in the large dead space created by the removal of a giant cyst (managed by a closed-suction drain), wound infection, paresthesia from cutaneous nerve injury, and hypertrophic scarring [2,9]. The recurrence rate can be minimized by ensuring complete excision of all cyst epithelium, including any fistulous tract to its superior terminus, and by performing surgery in the absence of active infection [1,5,10].

Histopathology and Malignant Transformation

Histopathologically, second BCCs are characteristically lined by stratified squamous epithelium; ciliated columnar (respiratory-type) epithelium and mixed epithelial types may also be found [2,7,11]. Subepithelial lymphoid tissue with follicle formation is a defining histological feature, with some authors considering the presence of both keratin and lymphoid tissue as obligatory criteria for the diagnosis [1]. Bocchialini et al. confirmed a cyst wall with lymphoid stroma covered by squamous epithelium without atypia in their case [11]. Alshihmani’s histopathological examination revealed a squamous-lined cyst with lymphoid infiltration, consistent with a BCC [4].

Malignant transformation within a second BCC is exceedingly rare. El Omri et al. note that only 15 cases have been published in the world literature, including four carcinomas in situ and 11 infiltrating squamous cell carcinomas [5]. Bellakhdhar et al. observed two cases of malignant degeneration among their 34 patients (5.9%), which were managed with cervicotomy, functional lymph node dissection, and adjuvant radiotherapy in one case [1]. The diagnosis of primary BCC carcinoma requires exclusion of cystic metastasis within the cyst from a distant primary tumor (particularly oropharyngeal squamous cell carcinoma) and a cystic lymph node metastasis [1,5]. The combination of in situ dysplasia and carcinoma confirmed within the same cyst is the fundamental criterion establishing primary malignant transformation [1].

Because of this malignant potential, the operative indication for confirmed second BCCs is universally considered formal, regardless of symptoms or patient age [1,5]. In the adult patient presenting with a lateral neck cystic mass, thorough exclusion of a primary head and neck malignancy with cystic metastasis, via FNA, CT, endoscopic examination of the upper aerodigestive tract, and, if indicated, PET-CT, should precede surgical planning.

The present case illustrates several important clinical principles in the management of giant second BCCs. First, the extraordinary size and bilobed configuration of this cyst (12 × 8 cm, extending from the skull base to the subclavicular region) far exceed the typical dimensions reported in the published literature, where the mean diameter is approximately 3 to 10 cm [6,10]. While El Omri et al. and Berrerhdoche et al. have each reported large second BCCs (8 cm and 65 × 50 mm, respectively) in young adult patients with uncomplicated courses [6,7], a bilobed cyst of this magnitude traversing both the posterior triangle and the inferior cervical compartment is uncommon.

Second, the rapid recurrence of the cyst within approximately two weeks of aspiration confirms the established principle that aspiration provides only temporary relief and is not curative [1,8]. The decision to proceed directly to surgical excision after documented recurrence was appropriate and consistent with the standard of care across all reviewed series [1,3,5-7,11]. Third, the operative strategy of controlled intraoperative decompression prior to en bloc excision was critical to managing this unusually large lesion safely. As supported by El Omri et al. and endorsed by Houck’s technical review [3,6], aspiration of a large cyst reduces mass effect and substantially improves visualization of the surrounding neurovascular structures, particularly when the cyst wall is in close proximity to CN XI, the carotid sheath, and the brachial plexus roots.

Fourth, the systematic identification and protection of CN XI was essential, given the superior lobe’s location deep to the nerve in the posterior triangle. Houck notes that the spinal accessory nerve must be identified and preserved during the second BCC excision, particularly for lesions with posterior triangle involvement, and that transient dysfunction may occur if traction injury is sustained [3]. Formal nerve identification with vessel loop retraction, as employed in this case, is the recommended technique to minimize this risk. Finally, the absence of intraoperative or postoperative complications, including chyle leak, nerve injury, hematoma, or wound dehiscence, reflects the importance of meticulous anatomical dissection, careful attention to the thoracic duct on the left side, and closed-suction drainage of the large dead space created by removal of a 12 × 8 cm cyst. These outcomes are consistent with the low complication rates reported in series where surgery is performed electively in the absence of active infection [1,6,11].

## Conclusions

This case demonstrates the successful surgical management of a giant bilobed second BCC extending from the skull base to the subclavicular region in a 24-year-old woman. Careful preoperative planning, controlled intraoperative decompression, and meticulous dissection with CN XI identification and preservation were essential to achieving complete excision without complication. Final pathology confirmed a benign second BCC with characteristic stratified squamous epithelial lining and subepithelial lymphoid follicles.

Clinicians should maintain a high index of suspicion for BCCs in young adults presenting with cystic lateral neck masses, as these lesions may attain considerable size before diagnosis. A correct initial diagnosis is critical, as incision and drainage substantially increase recurrence rates and complicate definitive surgical management. Complete surgical excision remains the gold standard, and early intervention before infectious complications develop optimizes operative conditions and minimizes recurrence.
